# Individual and collective factors influencing consumer attitudes and behaviour towards edible insects in Kinshasa: a pilot study

**DOI:** 10.1080/21642850.2023.2229411

**Published:** 2023-06-27

**Authors:** Nana Manwanina Kiumba, Olivier Luminet, Betty Chang, Emmanuel Mopendo Mwisomi

**Affiliations:** a University of Kinshasa, Kinshasa, Democratic Republic of Congo; b Psychological Sciences Research Institute, UCLouvain, Louvain-la-Neuve, Belgium; c Belgian Fund for Scientific Research (FRS-FNRS), Brussels, Belgium; d The European Food Information Council, Brussel, Belgium

**Keywords:** Edible insects, theory of planned behaviour, Africa, nutrition, food, alternative protein

## Abstract

**Background:** More than 300 peoples in the world consume edible insects either as a component of the traditional diet, or in the event of famine. Despite the benefits of insects, their acceptance by some consumers as a source of human food remains the main obstacle to their consumption. The present study focuses on the consumption of edible insects in the Kinshasa city (DRCongo) in a context of food crisis and shortage.

**Methods:** The study examined individual (attitudes, perceived control, intent); collective factors (subjective norms); context of consumption and emotional factors that influence insect consumption. A semi-directive interview study based on the theory of planned behaviour was conducted among 60 participants.

**Results:** The results showed that the consumption is a common practice in the study area, but that its frequency is influenced by factors related to the individual, such as participants’ positive attitudes towards insect consumption and ease of obtaining edible insects. The consumption of insects is also influenced by collective factors, such as family, friends, etc. The taste of insects, contextual factors such as family consumption, nutritional intake, habit and belonging to some specific tribes were related to greater consumption. Negative emotions, such as fear, insect characteristics or lack of knowledge about edible species were related to reduced consumption.

**Conclusions:** The results suggest that there is a need to implement interventions that focus specifically on changing certain attitudes.

## Introduction

Alternative proteins are receiving increased global attention. This burgeoning interest in plants (especially plant-based meat alternatives), insects, algae, and cultured meat has been attributed to their reported health benefits, lower environmental impact and improved animal welfare compared to conventional animal-based meat. Food producers and the media are promoting acceptance of these products, claiming superior nutritional, environmental and ethical credentials and a desirable novel sensory experience (Tso et al., 2020).

The consumption of edible insects is a very old practice in the world (Looy et al., 2014; Mignon, 2002). In Western countries, it has been gradually abandoned (Much, 2012). In some African countries such as the Democratic Republic of Congo (DRC), the consumption of edible insects is a practice that has been maintained to the present day because edible insects are among the most readily available natural sources of protein (Nsevolo et al., 2016).

The world population is growing very fast. The Food and Agriculture Organization of the United Nations (F.A.O., 2013) predicts that the world population will reach more than nine billion in 2050 and that most of this increase will occur in developing countries. In order to feed these populations, food production must increase by nearly 70% compared to current rates (Fouquet, 2015). However, land and energy resources are too limited to produce enough food to meet the growing demand of the population. Alternative sources of food must therefore be found. Edible insects are an interesting alternative because they contain protein and are rich in calcium, iron and zinc (Van Huis, 2012). In addition, they have a high feed conversion rate, which means that a proportionally smaller amount of food is needed to produce the same amount of protein, compared to the production of meat, for example (Van Huis, 2012). However, in spite of all these advantages, many barriers limit their consumption for reasons that we will explain in this article.

### Context of the study

The present study focuses on the consumption of edible insects in the DRC, more specifically in the city province of Kinshasa. Several studies have demonstrated the importance of consuming insects to overcome the scarcity of quality food in some regions of the world (Gallen & Pantin-Sohier, 2015). This includes the nutritional value of edible insects (Godfray et al., 2010; Okangola et al., 2016; Pal & Roy, 2014), their consumption frequencies (LrPayne et al., 2016), as well as psychological factors that may predict their consumption, including the role of individual (attitudes, intention, perceived control, emotions) and collective beliefs (subjectives norms) about edible insects (Gallen & Pantin-Sohier, 2015). To date, no studies have been conducted in the DRC to identify the individual and collective factors that influence consumer attitudes and behaviour towards edible insects. However, DRC is one of the few regions in the world where edible insects have been consumed for a very long time (Bomolo et al., 2017). The Congo Basin also hosts one of the richest reservoirs of edible insect species in the world (LrPayne et al., 2016). This study takes place in a context of war and conflict, and its associated disrupted transport network and supply chain.

In the context of economic and political crisis, food in the DRC takes on an essentially quantitative connotation. Households are turning to the least expensive and most high caloric products at the expense of protein-rich foods, resulting in unbalanced diets (Duquesne et al., 2010). This imbalance is exacerbated by an influx of imported products responding to new dietary practices of urban consumers such as increased consumption of meat and fish (Duquesne et al., 2010). To meet this increased demand for animal proteins and to reduce the consumption of imported products, the consumption of edible insects would be an essential alternative to improve nutritional protein intake in DRC.

### Status of edible insect consumption around the world

The consumption of edible insects is a practice found in all continents with a few Western countries (Mignon, 2002). In Africa, edible insects play a major role during the rainy season when the availability of staple foods such as fish and game decreases (Van Huis et al., 2013). In several tropical countries, some species are even considered as delicacies and are sold at relatively high prices in the markets (Van Huis, 2012). Among the country's many crops, many include insects in their dietary repertoire (LrPayne et al., 2016). Among the reasons put forward for the consumption of edible insects in the DRC are the feeding habits transmitted from generation to generation by ancestors, their taste, availability, or nutritional value (Food and Agriculture Organisation of the United Nations, 2014).

### Application of the theory of planned behaviour as a basic model

We considered the theory of planned behaviour (TPB), which is one of the most widely used and validated theories for predicting health-related behaviours and potential changes in them. It aims to predict and explain human behaviour using a combination of personal and social factors and is applicable to a variety of situations (Fishbein & Ajzen, 2010; Hagger et al., 2020), including food consumption (Armitage & Conner, 2010). It is based on the observation that individuals make reasoned decisions and that behaviour is the result of the intention to engage in the act (Hagger et al., 2020; Steg & Nordlund, 2013). TPB includes five main components. Firstly, attitudes represent a person's evaluation or judgment of a behaviour, either favourable or unfavourable (Fishbein & Ajzen, 2010). Subjective norms represent the social pressure perceived and experienced by the individual, whether from society in general or from those around him or her, to behave in a certain way. Subjective norms are based on the individual's normative beliefs, i.e. the behaviour that he or she believes to be the most acceptable given his or her social environment (Fishbein & Ajzen, 2010).

Perceived behavioural control refers to an individual's assessment of how difficult or easy it will be for them to perform a behaviour (Fishbein & Ajzen, 2010). Intention is understood as a deliberate will to perform an act or a disposition of mind in which one deliberately sets out to accomplish a goal. It is determined by the individual's attitudes, subjective norms, and perception of control over that behaviour (Fishbein & Ajzen, 2010). Behaviour is the dependent variable that is predicted by the other four factors; it refers to the set of actions an individual takes in response to the various stimuli that surround him or her (Fishbein & Ajzen, 2010). We hypothesise that all the dimensions involved in the TPB would influence insect consumption.

TPB has been mainly considered in a Western environment. In the context of insect consumption, there is only one study conducted in Africa (Kenya) that considered TPB (Pambo et al., 2016). One important question is therefore to examine whether the components of TPB would be relevant in the context of the present study that was conducted in another African country (DRC). TPB has never been used in DRC in the context of edible insect consumption. The aim is to test the relevance of the different components of the model, and examine the need to adapt it to a new cultural context.

In addition to the socio-cognitive determinants that are evaluated in the TPB, eating behaviour can be strongly influenced by our emotions (Berthoz, 2015; Macht et al., 2004). Some studies suggest that emotional variables predict behaviour above and beyond the dimensions of the TPB, making the overall predictive model stronger (Conner et al., 2013).

#### Barriers for the consumption of edible insects

In addition to factors that favour behaviours, there are also some obstacles. In relation to the consumption of edible insects, studies show that consumption does not depend solely on the objective or sensory qualities of insects, but also on the social representations (i.e. the image that the consumer has of the advantages or disadvantages of consuming edible insects) in a given population (Gallen & Pantin-Sohier, 2015). Negative emotions such as disgust and fear are potentially some important barriers (Rozin et al., 2008). A study conducted in Australia showed that the majority of people aged 60 and over were disgusted with the consumption of edible insects, with the strongest reactions apparent among the female interviewees. This disgust is explained by the incompatibility of this practice with their beliefs and cultural values (Myers & Pettigrew, 2018). Disgust was also indicated as a barrier to insect consumption in studies conducted in Germany and Italy (Orsi et al., 2019; Tuccillo et al., 2020). A very recent study showed that in Belgium, China, Italy, Mexico, and the US, aversion and disgust were the most important barriers that led to rejecting whole and processed mealworms in these populations's diets (Tzompa-Sosa et al., 2023). In addition, fear has been shown to discourage insect consumption, as demonstrated specifically by the tribe of Yombe in Kongo Central Province (DRCongo) (Balinga et al., 2004). These results suggest that some specific negative emotions may play a central role in predicting insect consumption in the population of another area in the DRC that is the focus of this study, i.e. Kinshasa.

Other important barriers to insect consumption include food neophobia or the reluctance to eat and/or avoidance of novel foods (Pliner & Hobden, 1992) and safety concerns (Woolf et al., 2019). Food neophobia (FN) describes problematic fear-based avoidance/restriction of novel foods (Selles et al., 2021). Some people feel reluctant to eat new types of food, such as insects they never ate before. This is associated with fear and feelings of insecurity. Orsi et al. (2019) identified food neophobia as a psychological and personality barrier to insect consumption in Germans. In Italy, food neophobia was one of the main reasons for not eating insects (Tuccillo et al., 2020). In Brazil, by contrast, the participants generally demonstrated a willingness to eat a novel food such as an insect-based cookie when it reaches the market (Cheung et al., 2021).

## Materiels and methods

### Participants

The sample consisted of 60 inhabitants of the city province of Kinshasa (in its urban and peri-urban areas) (34 males, the age range was between 14 and 73 years). A majority of them had a higher level of education (high school and university) (53.3%), 38.3% had a secondary level (secondary school) and 8.3% a primary level (primary school). Concerning their professional activities, 63.3% were employees, 30% were unemployed, and 6.7% belonged to other categories. The majority of participants lived on the outskirts of the city province of Kinshasa (56.7%), the others lived in the city centre (43.3%).

### Procedure

We used the survey method with the semi-structured interview technique to examine insect behaviour components and their determinants. The interview was based on the dimensions of TPB, on emotional aspects and habits related to insect consumption, and on knowledge regarding the context in which people eat insects. The format of answers varied across variables. In most cases, we adopted a free format and/or a dichotomic one (yes/no). For intentions only, we used a Likert type scale ranging from strong disagreement to strong agreement. The reason for this was that intention is the central predictor of behaviour, and thus we needed to have a more precise answer format. We transformed into a Likert scale for a good interpretation. In the following lines we specify the level of the transformed answers in the Likert scale. However, some questions, for instance those related to the type of insects eaten, used an open format in order to include an exhaustive list. An open format was also used for the types of emotions experienced. The survey was conducted in August during the dry season. Insects such as caterpillars and crickets are available during the dry season. Caterpillars, for example, are collected in early August. Consumers had to rely mainly on immediate experiences. This method has the advantage of minimising the risks for memory biases. At the moment of the study completion, there was no ethics committee at the faculty of Psychology of University of Kinshasa. In October 2022, we submitted an ethical form to the newly established ethics committee from the school of public health at the University of Kinshasa, that was also responsible for projects in the domain of psychology. The committee evaluated the written document and interviewed one author (EM). After these two steps, the project was formally approved by the committee on October 17, 2022, with the approval number ESP/CE/097B/2022, in a letter signed by the vice president of the committee.

### Measures

#### Attitudes

The participants were first asked whether they liked to eat edible insects (yes/no format). Attitudes were then measured through three items (e.g. general preferences, beliefs in negative or positive effects for health) with a free format of the answer.

#### Subjective norms

Subjective norms were measured through three items about the category of people who encouraged them to consume insects (free-form response). The first item is related to people with whom one feels comfortable eating insects; the second item is related to people who encourage insect consumption and the third item is related to people who discourage this behaviour. Participants were asked to name the categories of people involved for each item.

#### Perceived behavioural control

Perceived behavioural control was measured by four items reflecting the ease of eating edible insects, such as the extent to which it is easy for respondents to obtain insects in their environment (free-form responses). The first item dealt with the ease of finding insects, the second with the reasons that make insects unavailable; the third item with insects that are available all year round and the fourth with the types of permanent insects. There are some farms in the provinces of Grand Bandundu and Kongo Central where the ecology lends itself to intensive natural production. These farms ensure a regular supply of insects in these regions, without putting too much pressure on the environment through excessive consumption, which could adversely affect the quantity of insects available in the future.

#### Behavioural intention

Behavioural intention was measured through five items on 7-point Likert scales (ranging from 1 = strongly disagree to 7 = strongly agree) to assess participants’ intention to eat insects. The first item was related to the intention to consume edible insects, the second to the quantity to be consumed, the third to the frequency of insect consumption, the fourth to the types of insects to be consumed and the fifth to the intention to consume insects in other forms. One item was for example: ‘Do you intend to buy and eat the insects this week?’

#### Self-report behaviour

Self-report behaviour was measured by five items (free format answers). Items are related to the practice of eating edible insects; frequency of consumption; reasons for consumption and non-consumption; types of insects consumed and not consumed. For example, the item assessing practice of eating edible behaviours was: ‘Do you consume edible insects?’.

#### Emotions and habits

The participants were asked to report in an open format how they felt about eating edible insects. They were free to name as many emotions as they wanted. Participants were also asked to indicate whether or not eating edible insects was a habit for them. This last item was assessed with a yes/no dichotomic format.

#### Context in which the inhabitants consume edible insects

The consumption contexts were assessed by two items using 7-point scales (from 1 = strongly disagree to 7 = strongly agree). The items examined with whom participants eat insects and the places where they consume them (e.g. Where do you eat the insects and with whom?) ‘Do you consume insects often in a family setting?’.

## Results

### Component: attitudes

Almost all participants interviewed (98.3%) reported that they like to eat insects. Of the 98%, 53.3% liked eating insects and 45% liked eating insects a lot. Some positive effects of insects were mentioned, such as contributing to health (protein intake), preventing negative effects (malnutrition) on health and helping with food diversification. Despite these positive attitudes towards insect consumption, a few negative effects caused by certain edible insects were also cited by the respondents, such as health risks that were cited by 80% of the participants. Among these risks, we can mention itching in the throat when consuming crickets in excess, as well as diarrhoea for excessive consumption of caterpillars. Other negative effects were cited by only a small number of participants. A small number thought that there were no negative effects (10%). Allergies were one example of negative effects.

### Component: subjective norms

The people who encouraged participants most to eat edible insects were family members (15.3%), followed by friends, colleagues and fellow churchgoers (13.6%). Some indicated that they were not encouraged by anyone (11.9%), while others mentioned the medical profession (5%), other health professionals (3.4%), teachers (3.4%) and market vendors (1.7%). Note: Percentages are low because only one answer was possible. The main reasons cited for encouraging respondents to eat insects are related to habits (18.6%), maintaining health (15.3%), nutritional value (10.2%), social value (6.8%). or the fact that they eat specific insects like caterpillars with their teammates or spouses (5%). One of the reasons for encouraging consumption was its high social value (Table 1).

**Table 1.** Reasons for encouraging insect consumption.

	f	%
Habits	11	18.6
Maintaining health	9	15.3
Nutritional value	6	10.2
Social value	4	6.8
Influence of teammates or spouses	3	5
Experience	1	1.7
Taste	1	1.7

Note: f = frequency.

A very small proportion of participants reported being discouraged from eating edible insects: 6.8% were discouraged by some members outside their family, 5% by members of their own family, and another 5% by the ‘other’ category (people born in the city who are not used to eating insects, people who do not know about edible insects, and those who do not eat them). The reasons given for discouraging participants from eating insects were related to their habits and culture, lack of affinity with edible insects or personal reasons (Table 2).

**Table 2.** Reasons for discouraging insect consumption.

	f	%
Not in the habits and culture	6	10.2
lack of affinity with edible insects	4	6.8
Personal reasons	4	6.8
Damage to health	3	5
For fear	1	1.7
For lack of money	1	1.7

Note: f = frequency.

### Component: perceived behavioural control

The majority of those interviewed believed that it is easy to obtain edible insects (78%). Those who answered that it was difficult to obtain them cited the difficulty of obtaining insects on the market (61.8%), the periodicity of their availability (30.9%), cost (22.7%), other unspoken reasons related to people (15.5%), and lack of transportation for the traders who come to sell them (7.7%). Insects collected in the wild are first dried and then transported to markets to conserve them for as long as possible. The insects bought in the market were therefore dead. As for the question related to availability of edible insects, the majority (52%) reported that edible insects are not available year-round because they are periodic or seasonal.

### Component: intention

The majority of respondents intended to buy and eat edible insects the week they were interviewed (94.9%). Among the 94.9% of those who intended to consume; 8.5% intend to consume rarely, 16.9% sometimes, 3.4% not very often, 52.5% often, 13.6% very often. Those who intended to eat edible insects during our surveys had planned to consume four glasses or more per week (27%), 2–3 glasses (42%), 1–2 glasses (4%), 1 glass (21%) or only half a glass (6%). Note*:* 1 glass of Caterpillars *(Cirina forda) = *79.43 g; 1 glass of Caterpillars *(Imbrasia) = * 88.06 g; 1 glass of Termites *= *54.83 g (Ikonso Mwengi, 2020).

As for the frequency of consumption, 27% said they were prepared to consume insects more than twice a week, 25% indicated twice a week, 14.6% once a week and 33.4% that they would consume them once every two weeks. Among the types of insects that respondents intended to consume, the vast majority cited caterpillars (96%). The figures were then much lower for crickets (10%), larvae of orycts (8%), locusts (2%), termites (2%) and grasshoppers (2%). The reasons given by those who did not intend to eat edible insects were related to the change in dietary needs, a participant's known allergy, and the fact that the person was on vacation and only ate them at work.

As for being prepared to eat them in other forms (like in flour), the percentages are relatively low, since only 37.3% were ready for that possibility, and under certain conditions (related to taste, maintenance of nutritional value, actual processing of the insects), 23.7% could eat them unconditionally, 11.9% put forward other reasons, such as the fact that the taste of edible insects will be lost, chemicals will be added, it will not be possible to check whether they are actually edible insects that are being processed, etc. Finally, 3.4% responded that they are not ready to consume insects in another form.

Among the elements that could change participants’ intentions and prevent them from consuming edible insects, the first reason is related to health (47.5%) and refers to the case where such consumption could harm their health. On the other hand, 35.6% reported that nothing could prevent them from consuming edible insects. Other answers were rare, they were related to the risk of loss of flavour (8.5%), or to some food preparation methods of preparing them (5%).

### Component: behaviour

The majority, i.e. 97% of participants have always consumed edible insects, only one participant had never consumed them because it is not part of his culture's habits, and another participant had consumed them but had temporarily stopped eating them because of an allergy. Regarding the frequency of consumption of edible insects, the majority of participants reported that they ate edible insects on average 2 or more times during the previous week (46.55%), others ate them less than twice during the previous week (36.20%), and 8.62% ate them once or twice during the last month. The majority of people consumed edible insects for health reasons, others because of their nutritional value, still others because of their cultural habits and finally some consumed them because they liked the taste (Table 3).

**Table 3.** Reasons for eating edible insect

	f	%
Health reasons	17	28.3
Nutritional value	15	25
Cultural habits	13	21.7
Taste	10	16.7
Less time in cooking	3	5
Easy conservation of insects	1	1.6
Personal tolerance	1	1.7
Total	60	100

Note: f = frequency.

The reasons for not eating certain edible insects are related to taste (35.6%), emotional reasons (32.2%), personal reasons such as their beliefs (personal beliefs) about eating edible insects (22%), poor cooking (22%), reasons related to culture and habits (11.9%), lack of knowledge (10.2%) and health reasons (5.1%).

For the categories of edible insects that were appreciated, up to three insects were asked to be reported. We note that caterpillars were cited by 93.3% of respondents as one of their three favourite choices, grasshoppers followed with 37%, then larvae of orycts with 35%, crickets with 30%, termites with 23.3%, then locusts with 8.3%. In spite of these regular eating habits, the same edible insects are not appreciated by all. Some participants, for example, do not like to eat certain species of caterpillars (23.7%), crickets (22%), termites (18.6%), larvae of orycts (18.6%), red ants (13.6%), grasshoppers (8.5%), aquatic cockroaches (3.4%), butterflies (1.7%) and praying mantis (1.7%) (Table 4).

**Table 4.** List of insects appreciated and consumed and not appreciated and not consumed

Insects	Insects appreciated and consumed		Insects not appreciated and not consumed	
	f	%	f	%
Caterpillars	56	93.3	4	6.7
Grasshoppers	22	37	5	8.5
Larvae of orycts	21	35	11	18.6
Crikets	18	30	13	22
Termites	14	23.3	11	18.6
Locusts	5	8.3	–	–
Red ants	–	–	8	13.6
Aquatic cockroaches	–	–	2	3.4
Butterflies	–	–	1	1.7
Praying mantis	–	–	1	1.7

Note: f = frequency.

### Emotions

One important reason for not eating certain edible insects is related to the negative emotion (like fear) they elicit, which was reported by 32.2% of respondents; 6.7% indicated that they do not eat insects because of their customs of certain edible insects. Particpants even mentioned fearing being blamed by their parents for not consuming insects according to tradition (as it is a habit passed on from generation to generation).

### Context in which the inhabitants consume edible insects

The majority of participants felt comfortable eating edible insects with the family (74.6%). The percentage is much lower for eating outside the family (16.9%), or in both situations (8.5%). Almost all participants interviewed ate edible insects with other people (98.3%). They consumed them more in the family context than in other contexts. Consumption was mostly with family members (91.5%), with the other categories being much less frequent, namely friends (28.8%), and the ‘other’ category (15.3%), which included neighbours, the community, peers, and during celebrations. A very small number of participants indicated that they ate insects alone (5.1%) or with co-workers (3.4%). At this level, several answers were possible, which accounts for the fact that the total exceeds 100%.

In terms of consumption locations, the results suggest two main categories. People ate edible insects either in a private social context (family, friends, neighbours, etc.) for 42.4%, or in a public social context (party, event, restaurant, bar), for 57.6%. Finally, 97% of participants felt neither embarrassed nor ashamed to eat edible insects in the presence of others.

## Discussion and conclusion

This studied identified the individual and collective factors that positively or negatively influence consumer attitudes and behaviours towards edible insects in the DRC province of Kinshasa. The results showed that several factors related to consumer behaviour, subjective attitudes, intentions, and norms, and perceived behavioural control positively or negatively influence consumer attitudes and behaviour towards edible insects. We will first discuss possible reasons for higher levels of edible insect consumption before we turn to reasons for lower levels of edible insect consumption.

### Higher levels of edible insect consumption

The results showed that the large majority of the subjects interviewed, 97%, consume edible insects. These figures are higher than those published by Mapunzu (2002) which showed that only 70% of the interviewed Kinshasa inhabitants consume edible insects. The reasons for the difference with our study can be potentially explained by insect consumption becoming more popular over time, and/or by differences in the nature of the questions asked in both studies, or in the type of sample. Since the consumption of edible insects is a long-standing practice in the targeted study area, the first trend related to ‘consumption behaviour’ is impacted by several factors identified in this research. The reasons for eating edible insects are related firstly to health in general, then to the nutritional value of insects, then to cultural habits and finally for taste reasons.

About a quarter of the participants cited general health reasons. This may imply two complementary motivations, namely the desire to maintain their current state of health and avoid illnesses related to poor nutrition, and the desire to maintain or build their health because they consider insects as natural foods. Another quarter of the participants eat insects because of their nutritional value, knowing that insects contain proteins. Studies from Mapunzu (2002), Godfray et al. (2010), Pal and Roy (2014); Van Huis (2012) showed similar results. Approximately one-fifth consume them out of habit. Several families have been consuming edible insects since their ancestors and have passed on this culture from generation to generation. This observation is supported by the research of Bomolo et al. (2017) who explain that DRC is among the few regions in the world where edible insects have been consumed for a very long time. Consuming insects for maintaining the cultural identity has also been reported in Oaxaca, Mexico, where the entomophagy practice is deeply rooted (Hurd et al., 2019). Finally, less than a fifth consume them for hedonic reasons, i.e. their pleasant taste.

Some of these reasons for eating insects may be explained by other factors not examined in the study. One possibility is that peope have become accustomed to eating edible insects in the last decade. The city of Kinshasa has acculturated those who came from elsewhere but who did not consume insects outside of the city. Increased insect consumption can also be explained by the growing food crisis in Kinshasa, where people are turning to foods that were not consumed in the past in order to alleviate hunger. In addition, from Mapunzu (2002) to the present, many studies on edible insects and the awareness campaigns have been conducted, which may also have an impact on public awareness of edible insect proteins.

### Low levels of edible insect consumption

In this section, we will explain the different factors that negatively influence the consumption of edible insects and propose some actions to be taken to facilitate their acceptance. Among the most cited barrier to insect consumption is their high cost (22.7%). In relation to this difficulty, it is important to understand that the price of edible insects depends on the environment, availability, and quantity of insects on the market. The more edible insects are available and in greater numbers, the lower the purchase price will be. Thus, it will be useful to control for the permanent availability of edible insects (e.g. by protecting nature against the misuse of bush fires and the killing of host plants, or by the cultivation of insects).

A second obstacle participants reported in our study is related to periodicity or seasonality. According to FAO (2013), edible insects are available in the city province of Kinshasa only from September to December. It will thus be important to increase the production, conservation, and processing of edible insects to make them more available all year through.

A third obstacle is the disappearance of certain types of edible insects. The causes of this disappearance are bush fires, abusive harvesting, deforestation or abusive logging, other forms of degradation of the forest or host plants, etc. For this factor, the political authorities have an important role to play to prohibit, through the customary chiefs, the abusive use of bush fires and deforestation to allow insects to grow in safe environments. Very few participants cited factors that are related to allergies caused by certain edible insects, which cause symptoms such as diarrhoea, scabies, etc. But in order to encourage insect consumption, participants will also need to understand the reasons that could explain the allergy. Among the potential factors, we can cite undercooking.

Although the Theory of Planned Behaviour identified a set of relevant explanations for insect consumption, emotions felt in relation to entomophagy seems to be an additional determinant. In the present study, we identified negative emotions such as fear, disdain, repulsion, rejection, or anger. These negative emotions could be related to health issues that have been attributed to insect consumption. Another source could be lack of knowledge regarding the taste or the nutritional value of some insects. As regards to emotions about edible insects, psychologists have an important role to play in educating consumers on the benefits of entomophagy by exposing them to edible insects, which could help mitigate the negative emotions often experienced (Woolf et al., 2021).

The majority of the factors negatively influencing insect consumption that we have listed have also been identified by other studies, such Rozin et al. (2008) and Gallen and Pantin-Sohier (2015).

### Actions to be taken

In relation to the actions to be taken, consumers should be informed about the health and societal benefits of edible insects. For example, campaigns to promote edible insect consumption by making people aware of the types of insects and their nutritional values will help the population to benefit from information segmented according to the benefits of edible insect consumption.

### Limitation of the study

We have chosen only the inhabitants of the city province of Kinshasa. Although including the cultures of the different provinces, Kinshasa does not actually represent the entire western part of the DRC. The study is also limited by its sample size and the fact that the sample was not representative in terms of age, level of education, etc.

This study opened the way for a large-scale study in the south-west of the DRC (Kinshasa, Grand Bandundu and Kongo Central). The results indicated the low consumption of edible insects in the province of Kongo Central (Mopendo Mwisomi et al., 2023). These results then generated a qualitative focus group study to assess the cognitive and non-cognitive determinants of low consumption in Kongo Central. The results of these focus groups indicated low consumption in the cities and in the village of Kongo Central (Mopendo Mwisomi et al., 2023a), while the consumption was higher in town. These results justify the choice of the village and the cities as the two settings for the behaviour change interventions that was conducted a few weeks ago (Mopendo Mwisomi et al., 2023b).
